# Personality development in psychotherapy: a synergetic model of state-trait dynamics

**DOI:** 10.1007/s11571-018-9488-y

**Published:** 2018-06-04

**Authors:** Helmut Schöller, Kathrin Viol, Wolfgang Aichhorn, Marc-Thorsten Hütt, Günter Schiepek

**Affiliations:** 1 Institute of Synergetics and Psychotherapy Research, Paracelsus Medical University, Salzburg, Austria; 2 Department of Psychology, Ludwig Maximilians University, Munich, Germany; 3 University Hospital of Psychiatry, Psychotherapy, and Psychosomatics, Paracelsus Medical University, Salzburg, Austria; 4 Department of Life Sciences and Chemistry, Jacobs University, Bremen, Germany

**Keywords:** Psychotherapy processes, Personality development, State-trait dynamics, Computer simulation, Synergetics, Mathematical modeling, Computational systems psychology

## Abstract

Theoretical models of psychotherapy not only try to predict outcome but also intend to explain patterns of change. Studies showed that psychotherapeutic change processes are characterized by nonlinearity, complexity, and discontinuous transitions. By this, theoretical models of psychotherapy should be able to reproduce these dynamic features. Using time series derived from daily measures through internet-based real-time monitoring as empirical reference, we earlier presented a model of psychotherapy which includes five state variables and four trait variables. In mathematical terms, the traits modulate the shape of the functions which define the nonlinear interactions between the variables (states) of the model. The functions are integrated into five coupled nonlinear difference equations. In the present paper, we model how traits (dispositions or competencies of a person) can continuously be altered by new experiences and states (cognition, emotion, behavior). Adding equations that link states to traits, this model not only describes how therapeutic interventions modulate short-term change and fluctuations of psychological states, but also how these can influence traits. Speaking in terms of Synergetics (theory of self-organization in complex systems), the states correspond to the order parameters and the traits to the control parameters of the system. In terms of psychology, trait dynamics is driven by the states—i.e., by the concrete experiences of a client—and creates a process of personality development at a slower time scale than that of the state dynamics (separation of time scales between control and order parameter dynamics).

## Introduction

There are some basic assumptions in psychotherapy which seem to be evident: psychotherapy is a process evolving in time and psychotherapy intends to change personality. At second sight both assumptions are everything but trivial. The fact that human development is a dynamic process requires time series data in order to get an idea on what these processes look like. There is empirical evidence that doubts the linearity of human change processes and instead suggests discontinuity and nonlinearity (chaoticity) of the processes (Haken and Schiepek 2010; Hayes et al. 2007; Kowalik et al. 1997; Lutz et al. 2013; Schiepek et al. 1997, 2016a; Stiles et al. 2003; Strunk et al. 2015). In consequence, the challenge for the development of theoretical models on change processes is to explain nonlinear dynamics and discontinuous pattern transitions. Acknowledging that the explanandum should be both, the outcome and the process, mathematical algorithms are required which are able to create dynamics, e.g., computer simulations based on coupled nonlinear difference equations. Conceptually, this approach of modeling change dynamics is embedded in a meta-theoretical framework of nonlinear dynamic systems and self-organization (Haken 2004; Gelo and Salvatore 2016; Haken and Schiepek 2010; Orsucci 2006, 2015; Pincus 2009; Salvatore and Tschacher 2012; Schiepek et al. 1992, 2016a; Strunk and Schiepek 2006).

The second assumption on personality development is just as challenging as the nonlinear dynamics conjecture. The term ‘personality’ is a fuzzy psychological construct with different definitions, conceptualizations, and ways of operationalization. Early behavior therapists therefore neglected this construct and focused on observable (overt) behavior. In psychoanalysis, personality was part of the unconscious and its drive dynamics, based on early childhood experiences and only partially accessible to conscious experience and reflection. In psychology, personality is usually defined by traits in the sense of habitual patterns of behavior, thought, and emotion. According to this perspective, traits are relatively stable over time, differ across individuals, and influence behavior. States, in contrast, are conceptualized as transitory and fluctuating. The trait approach was based on Allport and Odbert’s work who clustered terms taken from an English dictionary that could be used to distinguish the behavior of one human being from that of another (Allport 1937). They differentiated between terms that represented general characteristics that determine personality—consistent and stable modes of an individual’s adjustment to his environment (traits)—and terms that referred to temporary experiences, moods, and activities (states). Cattell (1943) distilled Allport and Odbert’s trait terms into a useful taxonomy, and some decades later, the Big Five (Costa and McCrae 1992; Goldberg 1992) or the Big Six (Thalmayer et al. 2011) tried to capture the principal dimensions of human personality. Other models included the dynamics of personality development and the trans-situational variability of human’s thinking, feeling, and behavior (Magnusson and Endler 1977; Mischel and Shoda 1995). For example, Fleeson’s Whole Trait Model (Fleeson and Jayawickreme 2015) combines the evidence for interindividual differences in average global traits with the evidence that people also vary substantially around these averages. Consequently, they conceptualized personality traits as density distributions of momentary states. Based on this model, Wilson et al. (2016) tested, if fluctuations in affect and/or situational triggers account for fluctuations in personality states—measured in a sample of students by momentary ecological assessment—, finding that affect accounted for most, but not all of the within-person variance of states.

Other than in the Fleeson approach, the model of psychotherapeutic change we refer to in this article (Schiepek et al. 2017) differentiates in a classical sense between traits and states. The intention of the model is to reproduce some basic features of psychotherapy dynamics, like the variability of states, the evolution of state dynamics, but also the evolution of traits and the interaction between states and traits—in other words: the development of personality. The results we presented in previous publications focused on the dynamics of the model, e.g., nonlinear features and deterministic chaos, and on the dependency of the dynamic patterns (attractors) on the parameters—which can be interpreted as traits (Schiepek et al. 2016b, 2017)—, but did not consider the dynamic interaction between traits and states. Closing that gap is the aim of this article.

## The model

This model takes for serious that one of the most robust findings in common factors research is the importance of the client contributing to the course and outcome of psychotherapy (Bohart and Tallman 2010; Duncan et al. 2004; Orlinsky et al. 2004; Sparks and Duncan 2010; Wampold and Imel 2015). For this reason the model focuses on psychological mechanisms which have repeatedly been shown to be important within the “client system” both empirically and theoretically (e.g., Grawe 2004; Orlinsky et al. 2004). Another reason for focusing on these variables is their correspondence to the factors (subscales) of the Therapy Process Questionnaire (TPQ, Haken and Schiepek 2010), which is used in the routine practice of psychotherapy feedback (Schiepek et al. 2016c).

**Fig. 1:**
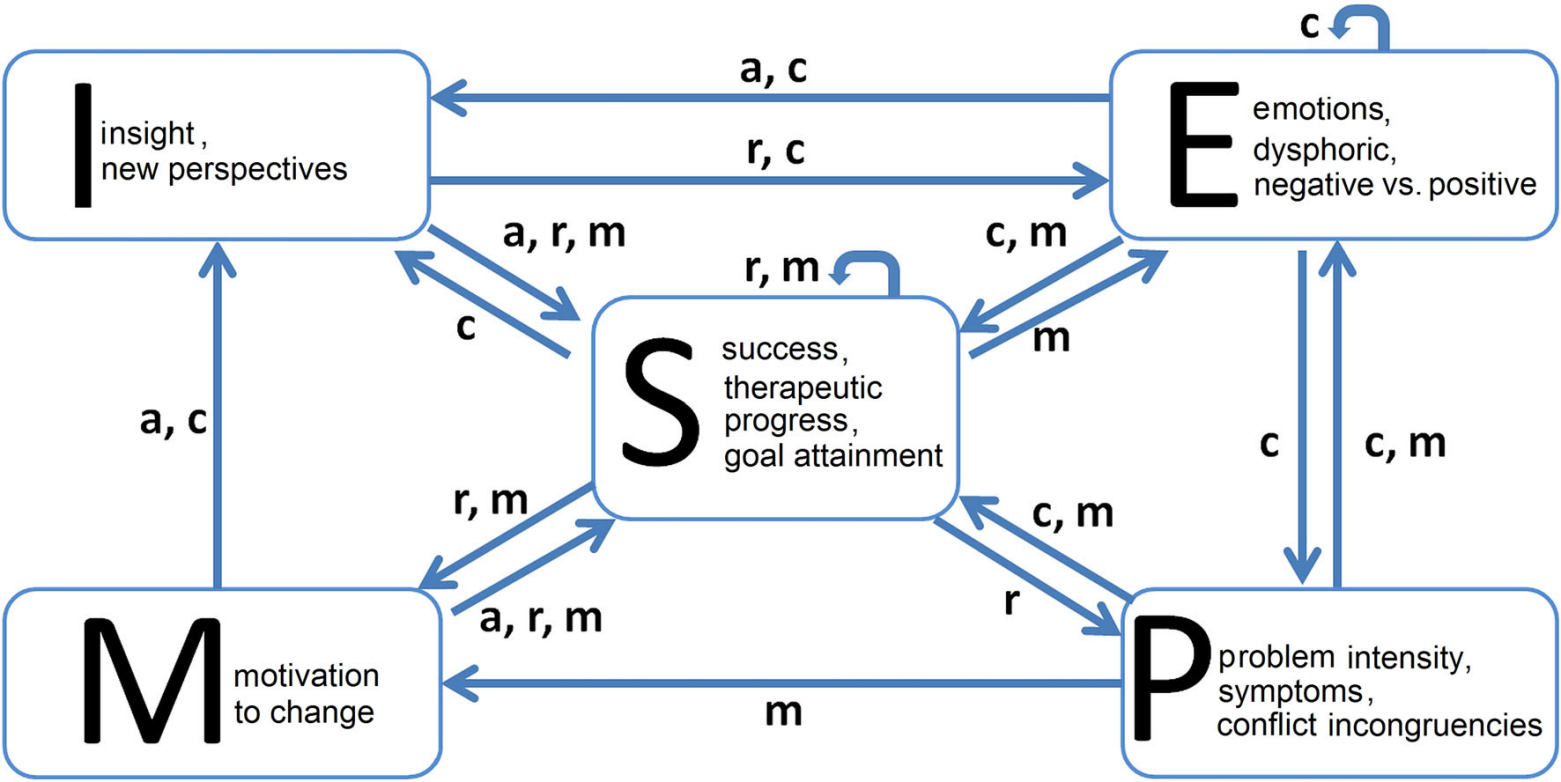
The structure of the model illustrates the dependencies between the variables and the parameters of the system

The model includes five variables which are connected by 16 functions, mediated by four parameters (Fig. 1). A detailed description of the constructs and the psychological mechanisms were given in Schiepek et al. (2017) and will be explained in more detail in a book which currently is in preparation. For a better understanding, a short description of the variables, parameters and functions will be given.

### The variables

(E) Emotions. This is a bidimensional variable representing dysphoric emotions (e.g., anxiety, grief, shame, guilt, and anger) at the upper end of the dimension (positive values of E) and positive emotional experiences (e.g., joy, self-esteem, happiness) at the lower end (negative values of E). This definition of polarity is based upon the results of a factor analysis of the Therapy Process Questionnaire (TPQ, Haken and Schiepek 2010), which is used to generate the empirical data for model testing.

(P) Problem and stress intensity, symptom severity, experienced conflicts or incongruence

(M) Motivation to change, readiness for the engagement in therapy-related activities and experiences

(I) Insight; getting new perspectives on personal problems, motivation, cognition, or behavior (clarification perspective in terms of Grawe 2004); confrontation with conflicts, avoided behaviors and cognitions, or with repressed traumatic experiences

(S) Success, therapeutic progress, goal attainment, confidence in a successful therapy course.

### The parameters

The model includes four parameters which mediate the interactions between variables. Depending on their values, the effect of one variable on another is intensified or reduced, activated or inhibited. Formally they modify the functions which define the relationship of the variables to each other.

(*a*) Working alliance, capability to enter a trustful cooperation with the therapist, quality of the therapeutic relationship, interpersonal trust. This parameter signifies the disposition to engage in a trustful relationship (attachment disposition) and also resembles the realized quality of the therapeutic alliance

(*c*) Cognitive competencies, capacities for mentalization and emotion regulation, mental skills in self-reflection, and the level of the personality structure (in the sense of the Operationalized Psychodynamic Diagnostics, www.opd-online.net)

(*r*) Behavioral resources or skills that are available for problem solving

(*m*) Motivation to change as a trait, self-efficacy, hopefulness, reward expectation, and “health plan” as suggested by the control mastery theory (Silberschatz 2009).

**Fig. 2:**
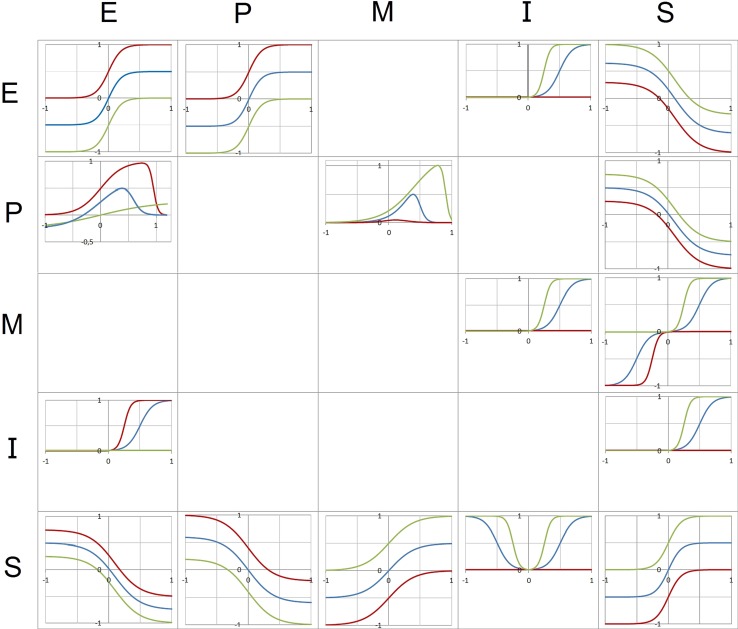
The figure represents the 16 functions of the model (for a detailed description see Schiepek et al. 2017). The variables noted on the left of the matrix (lines) represent the input, the variables noted at the top (columns) represent the output. Each function is represented by a graph in a coordinate system (x-axis: input, y-axis: output). Green function graphs correspond to the maximum of the respective control parameter(s) (= 1), red graphs to the minimum of the parameter(s) (= 0). Blue graphs represent an in-between state (0 < parameter value < 1)

The graphs in the coordinate planes of Fig. 2 illustrate how the shape of each function depends on the parameter values. The full range of the variables is covered by the functions defining the influence of other variables, that is, no arbitrary segmentations or thresholds have been introduced from the beginning. Thresholds and discontinuous jumps of the dynamics are emerging from the dynamics and not forced by some specific preliminary assumptions.

It should be noted that the variables and parameters are partially overlapping with the Research Domain Criteria (RDoC; Insel et al. 2010), promoted by the National Institute of Mental Health, which address similar psychological constructs, e.g., “negative valence” (variable E) or “attachment” (parameter *a*). Yet our model goes beyond the RDoC list by connecting the constructs into a large-scale model. Nonlinear dynamical models like the one proposed here are well suited to obtain this goal, not only by linking the elements but rather by formulating mechanisms of their interaction producing the emerging dynamics.

An empirical validation of the model is in preparation and will be based on 941 cases which were assessed (daily self-ratings) by the process questionnaire TPQ during the last years.

### The functions

The shape of each function represents theoretical as well as empirical findings from psychotherapy research (e.g., common factors research) and other psychological topics like emotion regulation, motivation, problem-solving and self-related cognition. The psychological interrelations between the variables were modelled by mathematical functions. Some connections are represented by functions of sigmoid shape and varying scales. The function $E_{t} \left( {S_{t - 1} } \right) = \frac{1.25}{{1 + e^{{5S_{t - 1} - 0.5}} }} - 0.5 - 0.5m$ for example describes how negative emotions E depend on therapeutic success S (Fig. 2, bottom left), i.e., the experience of negative emotions like fear, grief, shame, or anger are reduced or are inversely related to feelings of progress and being successful in solving personal problems, with a saturation effect for extreme values of S. The strength of the effect is mediated by parameter *m*, that is, by feelings of self-efficacy and a general positive expectation in problem-solving efforts. The higher *m*, the better S will reduce worrying emotions.

Other relations, e.g., E(P) and M(P), required more refined mathematical functions to capture the psychological mechanisms. The dependence of negative emotions E on the problem intensity P, for example, describes a complex relationship and represents the state of knowledge on emotion regulation and the psychopathology of borderline personality disorder (Fig. 2, left column, second from bottom). Increasing problems activate worrying and distressing emotions. The more severe or stressing the problem, the more such emotions will be triggered (exponential increase). This emotion triggering effect is more pronounced if the person has only minor competencies (red line) in emotion-regulation, self-reflection, and mentalization (parameter *c*) and/or reduced expectations in his/her capacity to solve problems or to manage difficult or stressful situations (self-efficacy expectation, parameter *m*). With higher values of in *c* and/or *m* (green line), coping strategies for the down-regulation of negative emotions at distinct problem intensities will be available and can be applied. The higher *c* and/or *m*, the lower the maximum of E and the earlier coping mechanisms and emotion regulation skills will reduce negative emotions. At low levels of *c* and *m* (red line), even lower levels of affect intensities cannot be managed or reduced until completely distressing and disturbing emotions (high levels of E) are interrupted, repressed, or disconnected from conscious experience by consuming drugs or alcohol, by self-harm, or by mechanisms of dissociation (switch of ego-states).

Finally, the functions are added to five coupled nonlinear equations, one for each variable, determining the dynamical system:

$$ \begin{aligned} E\left( {E,I,P,S,c,r,m} \right) & \quad = \frac{1}{{1 + e^{ - 10 E} }} - c + \frac{1}{{1 + e^{{ - 20 {\text{I}} \cdot \left( {1 - \frac{c + r}{2}} \right) + 5}} }} + \frac{{\frac{ - 1}{{1 + e^{{\left( {2 + 3 \cdot \left( {1 - \frac{c + m}{2}} \right)} \right) \cdot P}} }} + 0.5 + 0.5 \cdot \left( {1 - \frac{c + m}{2}} \right)}}{{1 + e^{{25 \cdot \left( {1 - \frac{c + m}{2}} \right) \cdot \left( {P - 0.2 - 0.75 \cdot \left( {1 - \frac{c + m}{2}} \right)} \right) }} }} \\ & \quad + \frac{1.25}{{1 + e^{5S - 0.5} }} - 0.5 - 0.5 m \\ \end{aligned} $$

$$ {\text{I}}\left( {{\text{E}},{\text{M}},{\text{S}},{\text{a}},{\text{c}}} \right) =  \frac{ 1}{{ 1 {\text{ + e }}^{{ - 20   {\text{E }} \cdot \left( {\frac{a + c}{2}} \right)   { + 5}}} }} + \frac{ 1}{{ 1 {\text{ + e}}^{{ - 2 0   {\text{M }} \cdot \left( {\frac{a + c}{2}} \right)   { + 5}}} }} + \frac{ 1}{{ 1 {\text{ + e}}^{{ - 2 0\cdot | {\text{S| }} \cdot c + 5}} }} $$

$$ \begin{aligned} {\text{M}}\left( {{\text{P}},{\text{S}},{\text{r}},{\text{m}}} \right) = \frac{1.261}{{    1 + {\text{e}}^{{ \left( {{\text{P}} - 0.05 - 0.85{\text{m}}} \right) \cdot \left( {10.1 + 19.9{\text{m}}} \right)}} }} \cdot \frac{1}{{1 + {\text{e}}^{{ - \left( {{\text{P }} - 0.43 + 0.03   {\text{m}}} \right) \cdot \left( {7 - 3m} \right)}} }} \\ & \quad - \frac{1}{{1 + {\text{e}}^{{  5{\text{S}}}} }} + \frac{{{\text{r}} + {\text{m}}}}{2} \\ \end{aligned} $$

$$ P\left( {E,S,c,r} \right)  =    \frac{ 1}{{ 1 {\text{ + e }}^{{ - 10   {\text{E}}}} }} - c + \frac{1.2}{{1 + e^{ 5S - 0.5} }} - 0.2 - 0.8 r $$

$$ \begin{aligned} S\left( {E,I,M,P,S,a,c,m,r} \right) & = \frac{1.3}{{1 + e^{5 E - 0.5} }} - 0.65 + 0.35 \cdot \left( {c + m - 1} \right) \\ & \quad + \frac{ 1}{{ 1 {\text{ + e}}^{{ - 2 0   {\text{I }} \cdot \frac{ (a + m + r)}{3}   { + 5 }}} }} + \frac{ 1}{{ 1 {\text{ + e}}^{{ - 2 0   {\text{M }} \cdot \frac{ (a + m + r)}{3} + 5}} }} - \frac{ 1}{{ 1 {\text{ + e}}^{{   2 0   {\text{M }} \cdot \left( {1 -  \frac{a + m + r}{3}} \right) + 5}} }} \\ & \quad + \frac{1.25}{{1 + e^{5P - 0.5} }} - 0.5 - 0.5 \cdot \left( {1 - \frac{c + m}{2}} \right) + \frac{ 1}{{ 1 {\text{ + e }}^{{ - 1 0   {\text{S}}}} }} + \frac{m + r}{2} - 1 \\ \end{aligned} $$

## Neural correlates of the phenomenological model

The variables and the parameters of this phenomenological model are defined at a psychological level, which of course is based on neuronal activity. Dating back to 1895, Freud made first attempts to link psychological processes to underlying neuronal mechanisms. It is worth noticing that he addressed the aim to link psychiatric disorders to the underlying neurobiological laws. More than a 100 years later, Kandel (1998) asked for a program on integration of cognition and behavior (especially related to psychiatric phenomena) with biological findings on brain processes. Since his seminal paper, the field developed rapidly and studies using different brain imaging methods (e.g., fMRI, EEG) revealed effects of psychotherapy on the activity of functional neuroanatomic structures and on neuronal networks (for reviews see Barsaglini et al. 2014; Schiepek et al. 2011). Research also focused on the brain mechanisms involved in therapeutic change processes (Cozolino 2010, 2015; Schiepek 2011).

Mathematical models were developed to explain the neuronal mechanisms of specific disorders. For example, a mechanistic framework of brain network dynamics underlying Major Depressive Disorder (Ramirez-Mahaluf et al. 2015) described how abnormal glutamate and serotonin metabolisms mediate the interaction of ventral anterior cingulate cortex (vACC) and dorsolateral prefrontal cortex (dlPFC) to explain cognitive and affective symptoms and its medical treatment by Selective Serotonin Reuptake Inhibitors (SSRI). Other approaches like The Virtual Brain (TVB; Leon et al. 2013; Ritter et al. 2013) integrate data from subjects (fMRI, MEG, or EEG) with full brain network simulations across different brain scales. TVB is a neuroinformatics platform for network simulations using biologically realistic connectivity which allows for the reproduction of a broad range of dynamic features, e.g., focal or distributed changes in the network dynamics of brain disorders and approaches to counteract those pathological processes.

Conceptually, simulations and measures at different brain scales focus on physico-chemical mechanisms which relate to mental or psychological phenomena (cognitions, emotions) like statistical mechanics of gas dynamics relate to phenomenological gas theory. In terms of Synergetics, we deal with a relative micro level of a multi-level and multi-scale system which may create order parameters at an emergent macro level (Haken 2002). Both levels are related to each other, but given our actual knowledge, there exist emergent qualities at the macro level (e.g., phenomenological consciousness) which cannot be fully reduced to the micro level. Anyway, the dynamics at two or more levels may be correlated (see the K model of Freeman 2000, 2004; Kozma 2016). In one of our own studies we were able to show that order transitions in the dynamics of cognitions and emotions during psychotherapy (assessed by daily self-ratings) were timely related to pattern transitions of brain activity (assessed by repeated fMRI scans; Schiepek et al. 2013).

A huge amount of neurophysiological studies investigated the neural underpinnings of the variables, parameters, and also the mechanisms behind the functions of our model. Any attempt to delineate these findings would be beyond the scope of this article. Especially the neurobiology of emotions (variable E) has created a neuro-psychological subdiscipline of its own: affective neuroscience. Also problem intensity (P) is related to the experience of stress and all neural and neuroendocrine mechanisms of stress regulation (Subhani et al. 2018).

Given the enormous amount of literature on the topic, only some findings should illustrate that the parameters of the model can be related to neuronal underpinnings. For example, the neuronal mechanisms of emotion regulation, which is an important part of the parameter *c*, concern the top–down regulation of the dorsal and ventromedial prefrontal cortex and of the ACC on limbic structures, including the insular cortex and the amygdalae as prominent regions (e.g., Etkin et al. 2015). Similar areas (e.g., the dorsal and medial prefrontal cortex) seem to be involved in mentalization (for a review see Mahy et al. 2014), justifying the combination of the two constructs in one parameter (c). The neuronal correlates of the parameter *m* have been investigated by Hashimoto et al. (2015). Based on the analysis of gray and white matter volumes, the authors suggest an internal locus of control, associated with self-regulation and reward expectation, encompassing the anterior cingulate cortex, striatum, and anterior insula. Dopaminergic structures such as the ventral striatum (nucleus accumbens), the putamen or the nucleus caudatus, are involved in reward expectation and motivation for goal directed actions (Knutson et al. 2001; Hurano and Kawato 2006). Krueger et al. (2007) found the paracingulate cortex and the septal area involved in partnership building and maintenance of reciprocal trust, comparable to the client’s engagement in the therapeutic alliance (parameter *a*). The modulation of neuronal activity by oxytocin and its receptor dynamics (Costa et al. 2009) relate to attachment styles as well as all neural networks recruited for empathy and theory of mind processes (Mahy et al. 2014) in interpersonal communication. These competencies together with behavior skills for social interaction and problem solving are concerned by the parameter *r* of our model.

## A synergetic interpretation of states and traits

The variables of the model can be understood as psychological states with varying intensities with a sampling rate of once per day, so that each iteration of a simulation run can be interpreted as a daily measurement of the variables. This corresponds to the way the TPQ is applied in practice. In terms of Synergetics, the variables represent the order parameters of the system. Order parameters are variables which describe the global bottom-up dynamics of a complex system. They are constituted by many sub-systems or sub-processes (e.g., the amplitude and frequency of convection cells in fluid dynamics, which are constituted by the molecules of the fluid), and also realize a top-down synchronization, which regulates (orders) the dynamic behavior of the sub-systems or system components (*enslaving principle*) (Haken 2004). Order parameters capture the most important information of a multi-component system on a few dimensions (*information compression*).

**Fig. 3:**
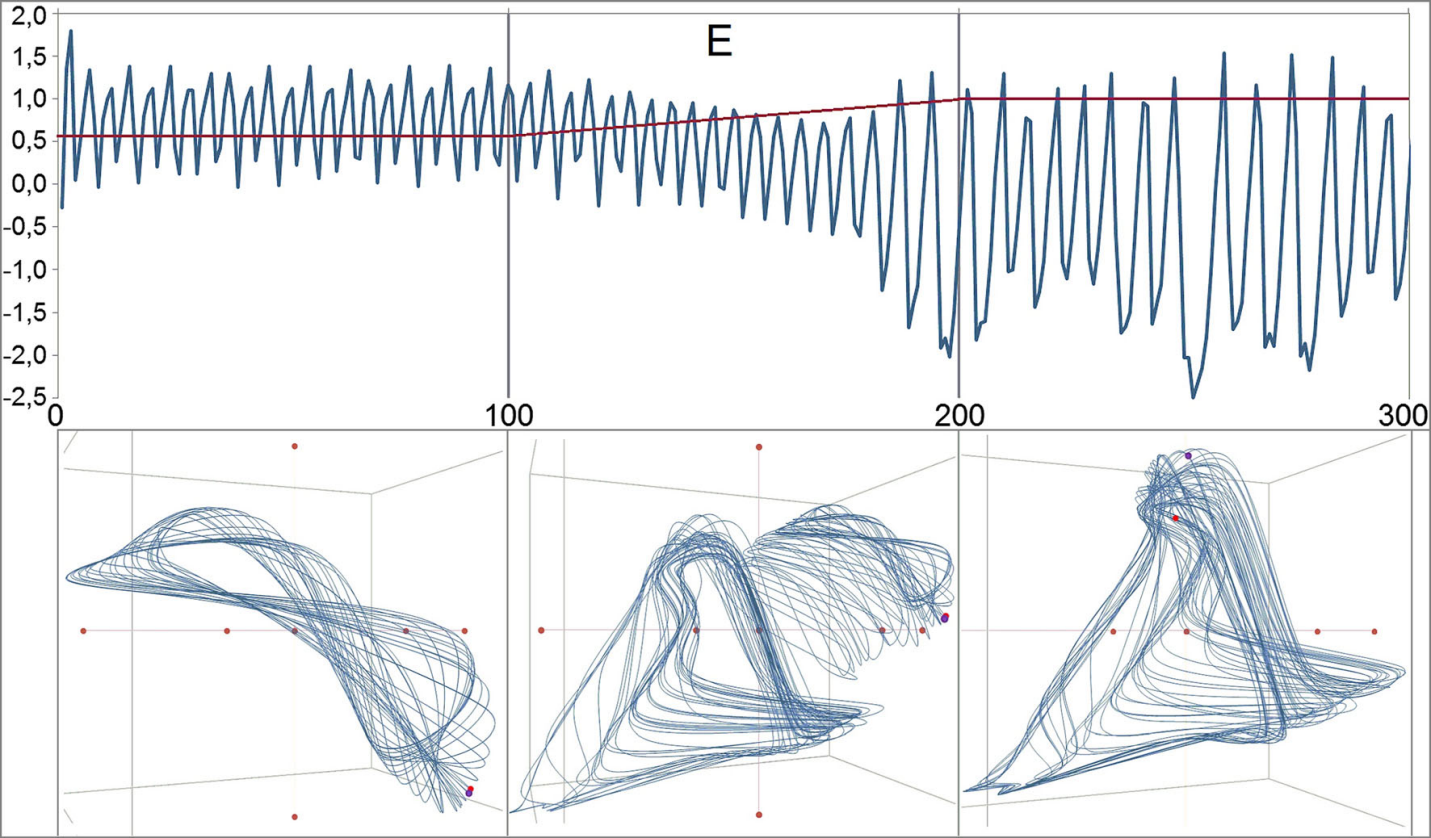
Order transition in the dynamics of the variable E. The numbers at the y-axis refer to the values of the parameter *c* (0 <* c *< 1, red line) and to the z-transformed values of E (blue line). The transition of the pattern depends on a stepwise linear increase of the parameter *c* from 0.60 to 1.00 between iteration 100 and 200. From iteration 0 to 100, the parameter is kept constant at 0.60, creating a certain dynamic pattern (attractor). After the 200th iteration, *c* is constant at 1.00, producing another pattern at a lower mean level of E, at a lower frequency, and with higher amplitudes of the chaotic oscillations. The attractors are shown below the time series. For the generation of the attractors, the discrete iterations were splined by the Excel standard spline function. During the linear stepwise increase of the control parameter, the transient attractor combines features of the pre- and the post-attractor and by this is more complex than each of both

While states correspond to the order parameters of the model, traits correspond to its control parameters. Psychologically, the control parameters can be interpreted as traits or dispositions changing at a slower time scale than the variables or states (separation of the time scales). In terms of Synergetics, the change of control parameters drives the phase transitions of the system (Haken 2004) (or in a more general and psychological sense the *order transitions*). Indeed, a linear and continuous change of one or more parameters may have sustainable effects on the dynamic patterns of a system, constituting a *phase transition* (Haken 2004). The effect of a parameter shift in *c* is demonstrated in Fig. 3. A continuous shift (continuous stepwise increase) in the sensitive range of the parameter produces a discontinuous jump of the system dynamics (order to order transition, Haken and Schiepek 2010).

**Fig. 4:**
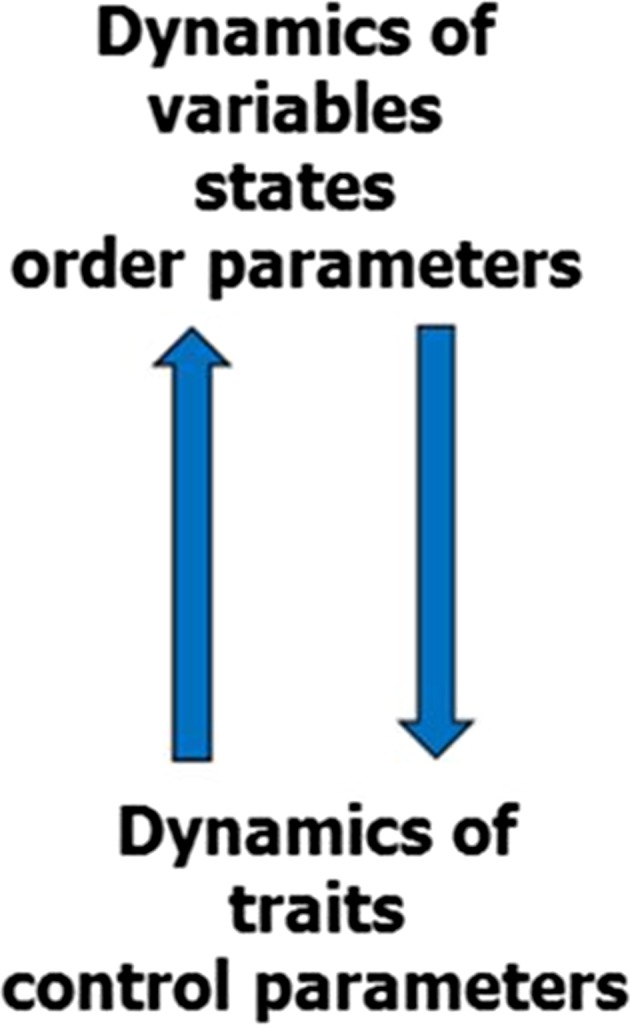
Circular causality between state (order parameter) and trait (control parameter) dynamics. The feedback-loop includes different time-scales

However, there is a big difference between control parameters in physical or physiological experiments, which are susceptible to direct external control (this is why they are called *control parameters*), and psychological parameters in the sense of traits. Traits are merely indirectly open to external input (Haken and Schiepek 2010). Traits in the sense of skills or competencies can be developed, but not directly influenced. They are dependent on concrete behavior, emotions, and cognitions, that is, on the experiences a person has in numerous consecutive specific situations. Any training program for skills or competencies uses such an indirect way of actualization of behaviors, feelings, and thoughts, that is, by the way of states (e.g., experiencing new behavior). *Learning* or *personality development* can in that view be expressed as the modification of the dynamics of a system by the modulation of the nonlinear functions that connect the order parameters with each other (states), while these states in themselves can modulate the traits or dispositions. There is a circular causality from traits to states and from states to traits, from control parameters to the order parameter dynamics, and from the dynamics of order parameters to control parameters (Fig. 4).

Allowing for a short historical side note, the fit of this conceptualization of personality development not only to Synergetics but also to other concepts of self-organization in psychology should be remarked. Especially the Gestalt psychology tradition goes back to the early twentieth century, when Gestalt psychologists Koehler (1920, 1940, 1947), Metzger (1940) and others described the emergence of patterns in perception, cognition, emotions, and behavior. In this paradigmatic frame, pattern formation is driven by basic psychological laws of “Gestalt”. These ideas were expanded by Lewin (1936a, 1936b, 1951), who included the impact of human needs, social contexts, and the personality on behavior. His topological view on personality integrated the environment as it is perceived by a motivated subject. The environment as a gradient field is given by the famous formula B = f (P, E): Behavior B is a function of the person P and his environment E. In this Lewinian tradition, the model proposed here is not aimed to describe averaged behavior for which statistics would be a suitable method, but focuses on the single case, that is, on the developmental trajectories of individual clients. Like in Lewin’s work, our model intends to explain psychological processes by mathematical means, nowadays called computational systems psychology.

## Model extension on parameter dynamics

The circular causality between states and traits demands an extension of the state or order parameter model described so far, which is realized as coupled nonlinear difference equations (discrete model with one equation for each variable, see Schiepek et al. 2017). The basic idea about the evolution of traits is its dependency on the increases or decreases of the states, i.e., concrete experiences in emotions (E), problem intensity (P), motivation to change (M), insight (I), and success (S).

Therefore, the functions describing the dynamics of the parameters *a*, *c*, *m* and *r* depend on the values of these variables. *a*_t_ depends on increases of success and on the experience of positive emotions. *c*_t_ depends on increased insight and on therapeutic success. Social and behavioral resources (*r*_t_) may also contribute to the evolution of *c*_t_, since these competencies may offer a broader range of personal experiences contributing to a better understanding of oneself and of one’s social environment. In the opposite direction, the evolution of *r*_t_ depends on cognitive competencies and on skills in emotion regulation (*c*_t_), which allow for a more effective development of social and other behavioral skills, together with success in problem solving and therapeutic progress in other fields. The evolution of self-efficacy, positive reward expectation and a generalized hopeful attitude to oneself (*m*_t_) depends on successful problem reduction, the experience of positive emotions, increased state motivation to change, and therapeutic success.

The influence of the state variables on the progression of the control parameters has to consider different time-scales for the variables’ evolution on the one and the trait dynamics’ evolution on the other hand (see the filter functions *f* in the parameter equations). Additionally, one has to prevent for favoring designated time-points, e.g., distinct starting values. Therefore, the most important effect on the parameters is exerted by the increase or decrease of the state variables in relation to a decay-affected mean value, and the actual values *a*_t_, *c*_t_, *r*_t_, *m*_t_ of the parameters at time t are calculated by functions which increase or reduce the parameter values of the last iteration *a*_t–1_, *c*_t–1_, *r*_t–1_, *m*_t–1_ to a certain amount—dependent on the long term impact of variable dynamics:

1 $$ a_{t} = a_{t - 1} + s_{a} \cdot w_{a} \cdot a_{t - 1} \cdot \frac{1}{2}\left( {f_{S,t,n} - f_{E,t,n} } \right) $$

2 $$ c_{t} = c_{t - 1} + s_{c} \cdot w_{c} \cdot c_{t - 1} \cdot \frac{1}{3}\left( {f_{I,t,n} + f_{S,t,n} + r_{t - 1} } \right) $$

3 $$ r_{t} = r_{t - 1} + s_{r} \cdot w_{r} \cdot r_{t - 1} \cdot \frac{1}{2}\left( {f_{S,t,n} + c_{t - 1} } \right) $$

4 $$ m_{t} = m_{t - 1} + s_{m} \cdot w_{m} \cdot m_{t - 1} \cdot \frac{1}{4}\left( { - f_{E,t,n} - f_{P,t,n} + f_{M,t,n} + f_{S,t,n} } \right) $$

Each equation consists of several elements that will now be explained in detail:

- *f*_*E,t,n*_*, f*_*P,t,n*_*, f*_*M,t,n*_*, f*_*I,t,n*_*, f*_*S,t,n*_ are filter functions which represent the effect of each variable on the respective parameter considering the differing time-scales by a combination of averaging and weighting recent changes stronger than prior ones. Within a running window of time length *n* (for the simulation runs of this paper, *n *= 14) the impact at *t*_*i*_ of the value depends on the sum of all differences from the arithmetic mean of the variable within the window, e.g., $\mathop \sum \limits_{i = 1}^{n} \left( {E_{i} - \overline{E} } \right)$. Using this procedure, not the absolute level of the variable has an effect, but its relative increases or decreases. In addition, we assume a memory effect which accentuates recent emotions or cognitions more than older ones. This is modeled by an exponential decay function with a characteristic steepness λ from the latest value within the running window (at *t*) to the oldest value at *t*–*n*. The exponential decay of the impact of each variable on the parameter change is given by $e^{{ - \lambda \left( {t - n + i} \right)}}$.
  The filter functions for the variables are given by expressions like this (here illustrated by E):
  5 $$ f_{E,t,n} = d_{E} \cdot \mathop \sum \limits_{i = 1}^{n} \left( {E_{t - n + i} - \overline{E}_{t,n} } \right) \cdot e^{{ - \lambda \left( {t - n + i} \right)}} $$
  In order to correct for the mean shift, which results from using decay-affected difference-values within the running window, correction factors (*d*_*E*_*, d*_*I*_, *d*_*M*_, *d*_*P*_, *d*_*S*_) are introduced. Their values are *d*_E_ = *d*_P_ = *d*_M_ = *d*_I_ = *d*_S_ = 0.535, for the decay-constants λ they are calculated from half-life constants ${{\uptau }}_{\text{E}} = {{\uptau }}_{\text{P}} = {{\uptau }}_{\text{M}} = {{\uptau }}_{\text{I}} = {{\uptau }}_{\text{S}} = 7{\text{d}}$, using the relation λ $= \frac{ln2}{\tau }$, resulting in ${{\uplambda }}_{\text{E}} = {{\uplambda }}_{\text{P}} = {{\uplambda }}_{\text{M}} = {{\uplambda }}_{\text{I}} = {{\uplambda }}_{\text{S}} = 0.099$.
- *w*_*a*_, *w*_*c*_, *w*_*r*_, *w*_*m*_ are weights which are introduced in order to dampen the effect of the variables on the parameters, i.e., scaling them to an appropriate range respective to the variables. They model the sensitivity and the impact of the state dynamics on the evolution of the traits. For the simulation runs presented in this paper, *w*_*a*_= *w*_*c*_= *w*_*r*_= *w*_*m*_ = 0.004167.
- The constants 1/2, 1/3 and 1/4 normalize the sum of contributors of the filter functions (may it be variables or parameters) to 1.
- The functions $s_{a}^{*} , s_{c}^{*} , s_{m}^{*} , s_{r}^{*}$ are saturation functions, which limit the growth or the reduction of the parameters onto the predefined range from 0 to 1. For example, the saturation of the parameter *c* is realized by
  6 $$ s_{{c_{t} }}^{*} = k \cdot \Delta c\left( {\frac{{1 + sgn\left( {\Delta c} \right)}}{2}\left( {c_{max} - c_{t - 1} } \right) + \frac{{1 - sgn\left( {\Delta c} \right)}}{2}\left( {c_{t - 1} - c_{min} } \right)} \right) $$
  - *k* is a gain factor for a windowing procedure, which restricts the possible range of the parameters [0,1] to the range of complex or chaotic dynamics, as it was defined by inspection of the bifurcation diagrams of the system (see Fig. 6 in Schiepek et al. 2017). For example, restricting *c* to the interval $0.1 \le c \le 0.8$ yields $k = c_{max} - c_{min} = 0.7$.
  - ∆c is the difference between c_*t*–*1*_ and c_*t*_.
  - The first term within the bracket is activated only if there was an increase in *c*: if ∆*c *> 0 → sgn(∆*c*) = + 1 → $\frac{{1 + sgn\left( {\Delta c} \right)}}{2} = 1$. For a decrease ∆*c* < 0 → sgn(∆*c*) = – 1 → $\frac{{1 + sgn\left( {\Delta c} \right)}}{2} = 0$. With the same logic, the second term is activated (unequal to zero) if there was a decrease in the parameter.
  - Furthermore, the saturation functions are activated only if the parameter values are beyond a certain threshold, > 0.8 or < 0.2 for all parameters. Taken *c* as an example:
    7 $$ s_{c} = \left\{ {\begin{array}{*{20}c} {\left( {1 - c} \right)s_{c}^{*}   | (c > 0.8) \cap (\Delta c > 0)} \\ {s_{c}^{*}    |  0.2 \le c \le 0.8} \\ {\left( {c - 1} \right)s_{c}^{*}    | (c < 0.2) \cap (\Delta c < 0)} \\ \end{array} } \right. $$
- Concerning the evolution of the parameter *a*_*t*_, the two aspects of parameter *a* can be taken into consideration. As we noted above, this parameter signifies the disposition to engage in a trustful relationship (attachment disposition). In the psychotherapy process, it also refers to the empirically realized quality of the therapeutic relationship between patient and therapist. In many studies, the therapeutic alliance has been proven as an important contributor to the therapeutic success (e.g., Flückiger et al. 2012; Wampold and Imel 2015). The alliance as perceived by the client can be measured by the items of the therapeutic alliance subscale of the TPQ. Hereby, the time series of the experienced quality of the therapeutic alliance of the psychotherapeutic process is hereby available. The concrete value of the empirically given quality of the alliance at time *t* is denoted *b*_*t*_. The two aspects are combined by calculating their mean,
  8 $$ a_{t}^{{\prime }} = \frac{1}{2}\left( {a_{t - 1} + b_{t} } \right) . $$
  Here, $a_{t - 1}$ is substituted by $a^{{\prime }}$, the mean of $a_{t - 1}$ and $b_{t}$. If no information is available about the values of $b_{t}$, they are set to $b_{t} = a_{t - 1}$ and therewith $a^{\prime} = a_{t - 1}$ in Eq. (8).

The interactive simulation system, performing simulation with the described framework and settings, can be used on www.psysim.at.

## Results: model dynamics

**Fig. 5:**
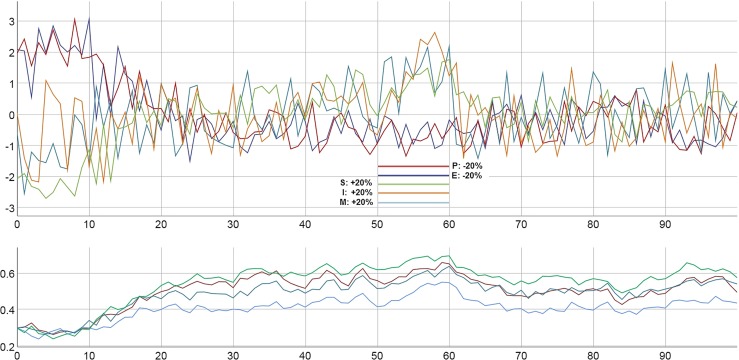
Noise-driven order transition between the 10th and the 20th iteration, accompanied by an increase of all parameters. Between the 50th and the 60th iteration, a multiple intervention is introduced (+ 20% on M, I, and S, − 20% on E and P). After this period, a spontaneous deterioration occurs since the effects of the interventions do not sustain. Parameters: *a*: red, *m*: green, *c*: bright blue, *r*: dark blue. Initial values: E: 97.6, P: 61.5, M: 7.5, I: 100, S: -40.7; all parameters: 0.30. Dynamic noise 30%, continuously. Variables: z-transformed. For this and the following figures, the respective simulations and simulation data are available for both download and direct application with our online simulation tool PSYSIM (www.psysim.at). We provide two types of links: with links named SIM-xx, you can open our online simulation tool PSYSIM and load the input and output of the simulation applied to the actual figures for direct inspection and further processing. Result data can be downloaded in CSV formal by the links named CSV-xx. SIM-5, CSV-5

**Fig. 6:**
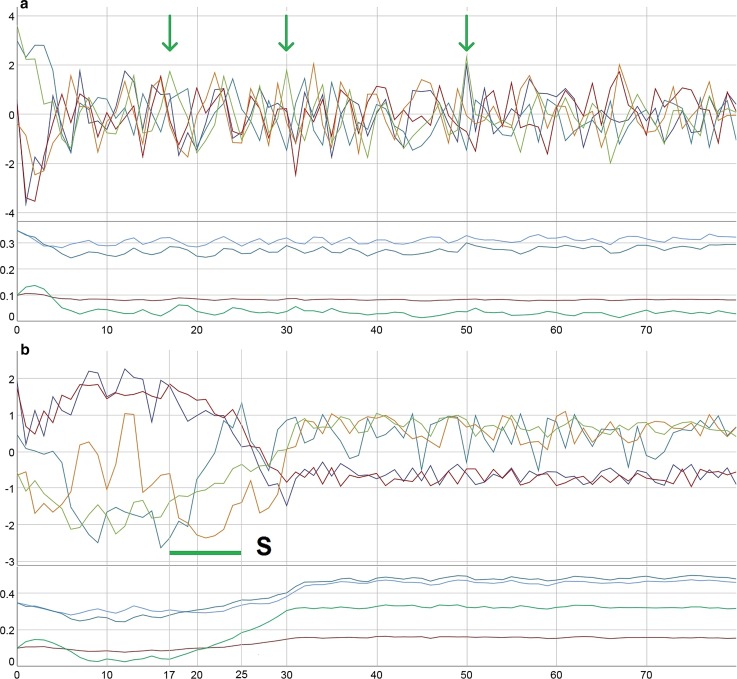
**a** Punctual interventions on S (+38%) at *t* = 17, 30, 50. Data: SIM-6a, CSV-6a. **b** Continuous interventions on S (+ 38%) from *t *= 27 to 25. Parameters: *a*: red, *m*: green, *c*: bright blue, *r*: dark blue. Initial values of variables and parameters: E: 100, P: 79, M: 32.5, I: 50, S: 33.5; *a*: 0.10, *c*: 0.35, *r*: 0.35, *m*: 0.10. Dynamic noise 10%, continuously. Variables: z-transformed. Data: SIM-6b, CSV-6b

**Fig. 7:**
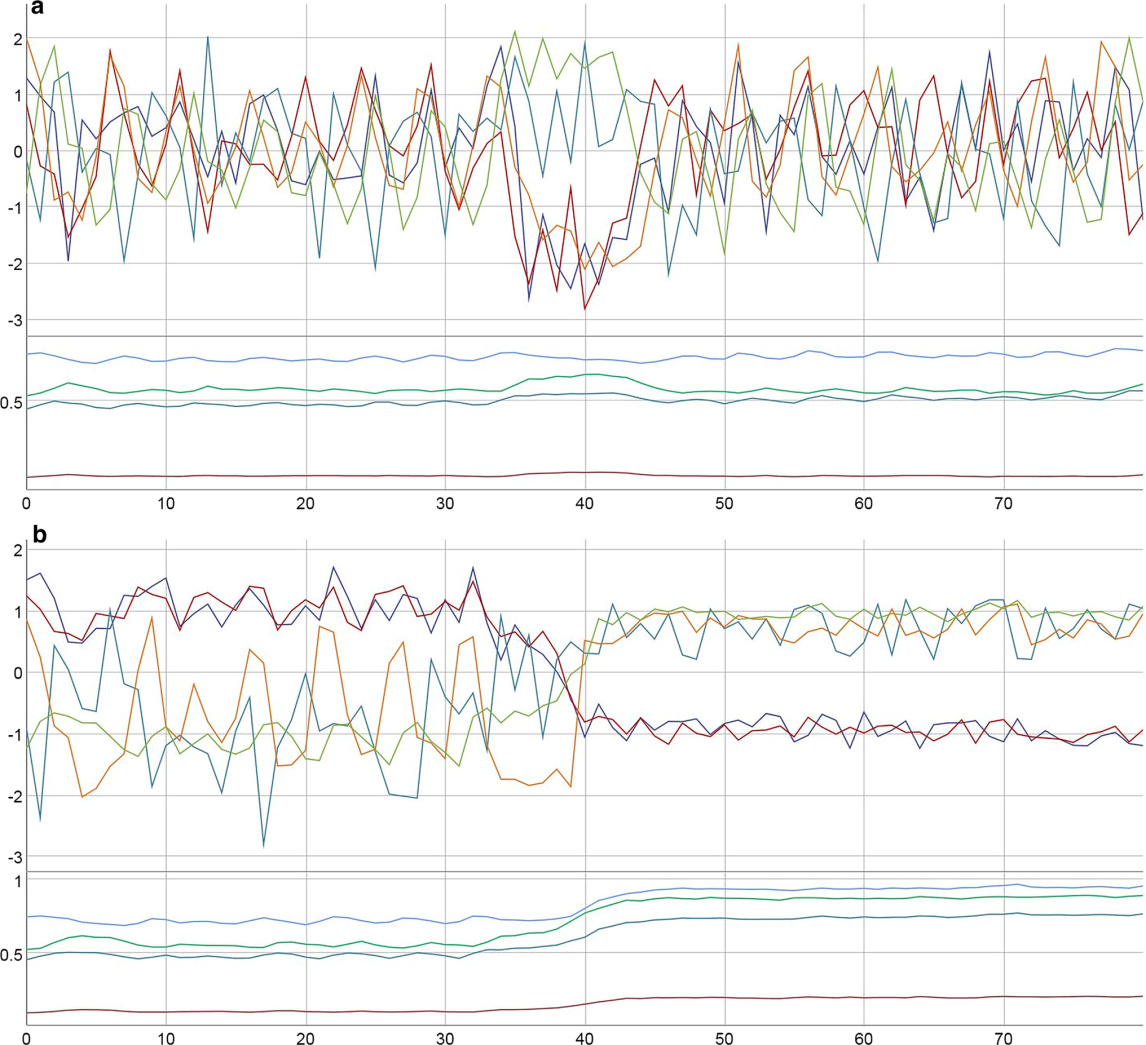
Two realizations (random numbers) of the same levels of dynamic noise (**a**, **b**). Parameters: *a*: red, *m*: green, *c*: bright blue, *r*: dark blue. In both cases, the initial values of variables and parameters are: E: 97.6, P: 61.5, M: 7.5, I: 100, S: − 40.7. *a*: 0.10, *c*: 0.75, *r*: 0.46, *m*: 0.53. Dynamic noise 10% on E and P, 5% on M, I, and S, continuously. Variables: z-transformed. Data: SIM-7a, CSV-7a, b: SIM-7b, CSV-7b

**Fig. 8:**
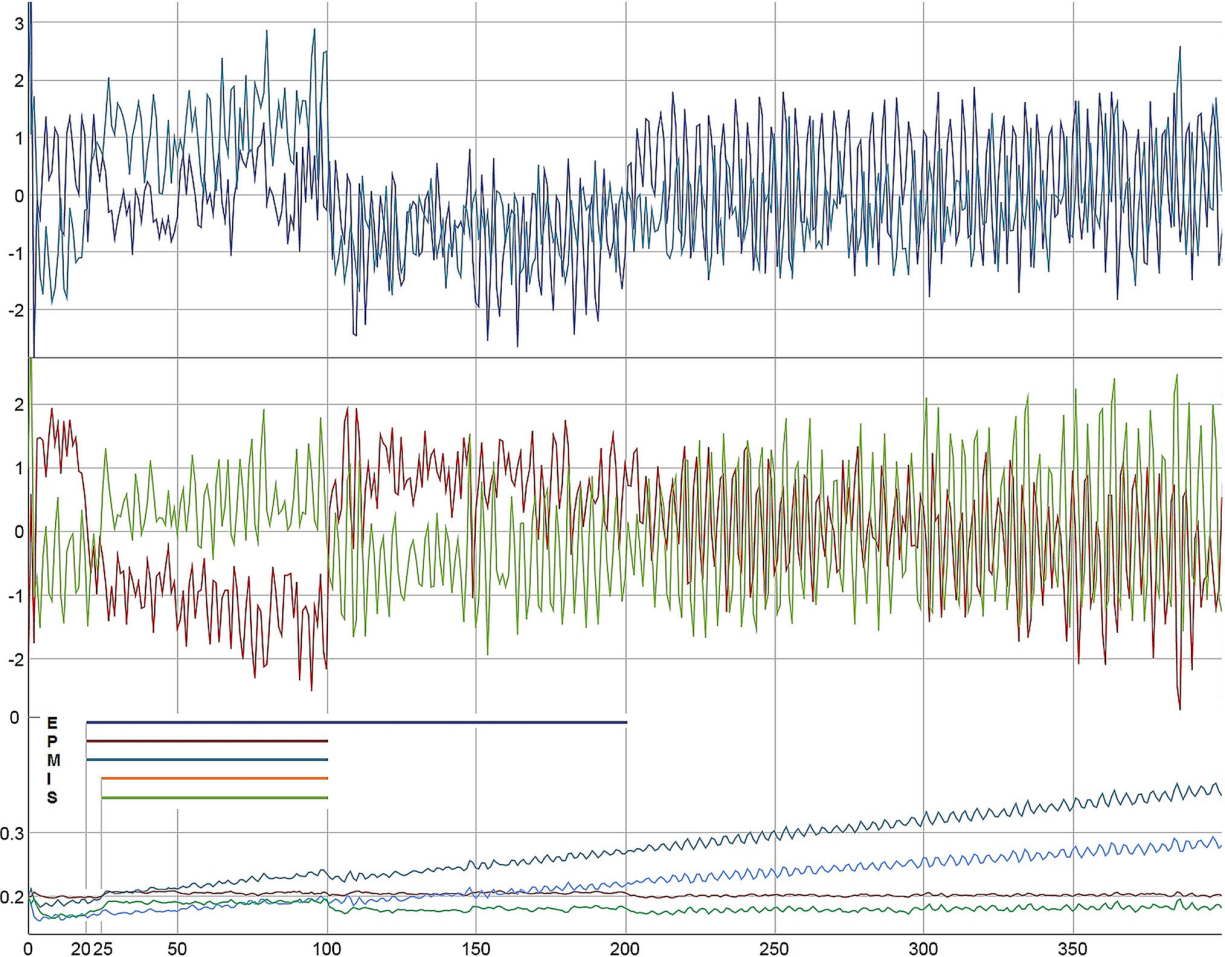
Interventions on E, P, and M start at *t* = 20, interventions on I and S at *t* = 25 (+ 5% on M, + 10% on S and I, − 10% on E and P). Except for E, all interventions end at *t* = 100, the intervention on E continues to *t* = 200. The interventions have an effect on all variables, but also a distinct rebound effect in S and M (decreases) and P (increase) can be observed. The continued intervention on E (− 10%) until *t* = 200 reduces stressful emotions, but also the motivation to change (M) (upper part of the figure). After this period, M and S increase slowly, and P decreases. It seems that a long-term recovery and self-healing process can only start if negative emotions are not suppressed, that is, the self-organizing effect onto another stable attractor can only take place if the system can follow its own unrestricted dynamics. Initial values of variables and parameters: E: 97.6, P: 61.5, M: 7.5, I: 100, S: − 40.7; *a, c, r, m*: 0.20. Dynamic noise: 2%, continuously. Variables: z-transformed. Data: SIM-8, CSV-8

In the following, some specific results of the simulated system behavior are presented. The simulation dynamics which are shown in Figs. 5, 6, 7 and 8 represent some characteristic features of the system and of psychotherapeutic processes. The dynamic patterns are based on specific parameter values and initial conditions, but can also be generated by other simulation runs within a range of parameter values and seed keys. Even without any specific interventions, unspecific dynamic noise applied to the variables can lead to a positive trend of the parameters (Fig. 5): a spontaneous transient period is realized at the beginning, from high levels of E and P and low levels of S and M to a balanced dynamics of all variables. Evidently, without intensive or continuous stressors or bad experiences, the model is capable of realizing a trend, which in psychological terms might be interpreted as a personal growth or self-actualization. On the long term, this could lead to spontaneous remission.

Interventions, which were implemented between *t *= 50 and *t *= 60 on all variables, have a time-limited impact on the state dynamics and by this, also on the traits. However, an order transition is not triggered by these multiple interventions.

Punctual interventions are less likely to change attractors than continuous evolution. In the example of Fig. 6a, the interventions on S (+ 38%) at *t *= 17, 30, and 50 have no impact on the dynamic pattern, and the parameters do not change neither except for small fluctuations around a stable state. However, longer periods of continuous intervention—in Fig. 6b an intervention of + 38% on S from *t *= 17 to 25 is applied—have a higher probability to change patterns. The existence of bi- or multistability in the dynamics of a system opens the option of order transitions with parameter drifts following the state dynamics, not only, as classical Synergetics predicts, from parameter drifts to order transitions.

Interestingly, sometimes unspecific daily hassles or spontaneous happiness, represented in the simulation as dynamic noise, can trigger order transitions. In Fig. 7a, a noise level of 10% on E and P and 5% on M, I and S has no long-term effect and qualitative impact on the dynamics (although from *t *= 35 to 45 a successful period occurs by chance). The same amount of noise, but with different random values, can trigger an order transition with long-term consequences on the trait levels (Fig. 7b). Here—like in Fig. 6b—the parameter drift seems to follow the state dynamics and to be a consequence, not a cause of the order transition. A closer look on the dynamics reveals a circular causality during the transition period: small changes in the levels of the variables (here due to noise) increase the level of the parameters, i.e., the client integrates new qualities of his/her experience and continues with higher competencies. This in turn affects his/her experience, represented by “better” values of the variables, until a new stable state is reached. From there, small perturbations (noise) cannot shift the system any further; the variables and parameters fluctuate around a certain fix point.

In many cases, a rebound effect occurs after a longer period of interventions. Correspondingly, many patients in real therapies indeed experience the release from inpatient treatment or from a day treatment center as a difficult time. Figure 8 illustrates this rebound effect: all interventions on P, M, I and S are stopped at *t *= 100. Only a reducing effect on stressful emotions of −10% continues, what might correspond to a continued intake of antidepressant or anxiolytic drugs. The continued (e.g., pharmacological) effect on E does not prevent the rebound effect to elevate the system to the same level and the same pattern as in the beginning, before any intervention had been started. Moreover, it seems to prevent a self-organizing process which on the long term relaxes the dynamics on a different “healthy” attractor. But continuously and especially after the intervention on E was stopped, a positive development in success and on problem reduction takes place, corresponding to an increase in competencies of *m* and *c*. In the example of this simulation run, but also in many others (not shown here), the model realizes a rebound effect to levels lower than at start. In the long run, both—state- and trait-dynamics—evolve to patterns that entail improvement (recovery).

**Fig. 9:**
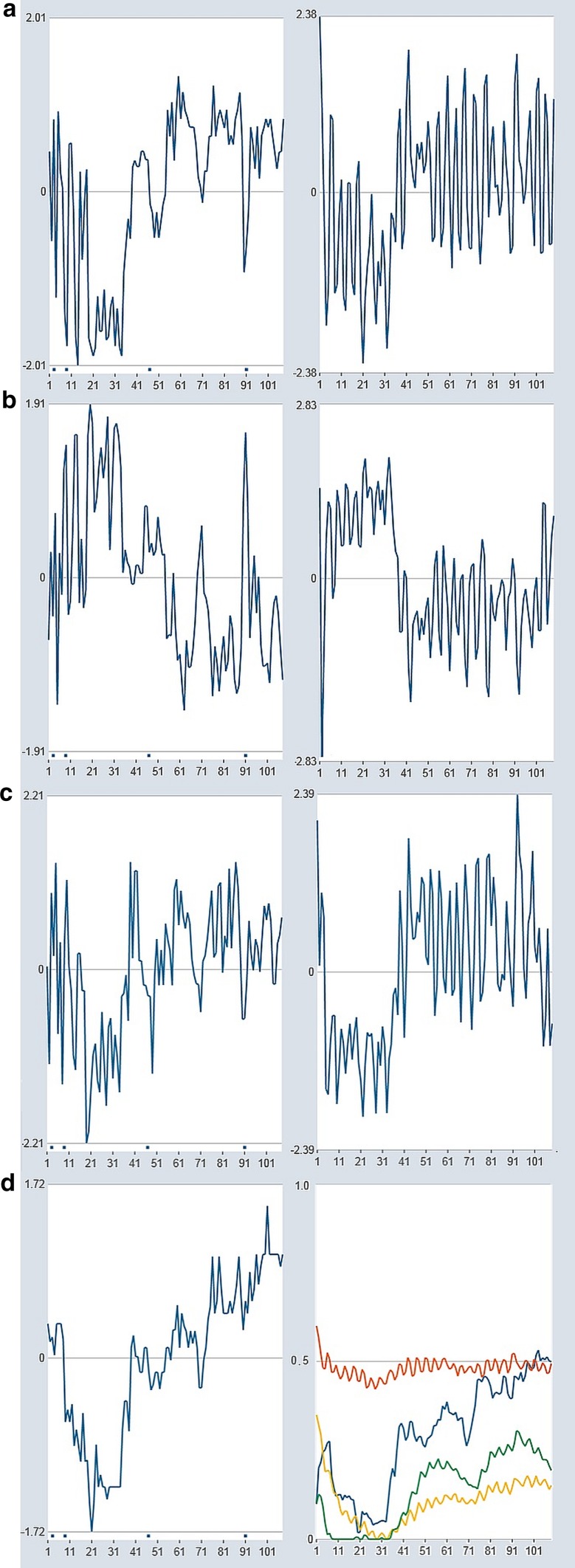
**a** Dynamics of the factor “Therapeutic Progress and Self-Confidence” of the TPQ as it was assessed by daily self-ratings (corresponding to S) in the real client (*t* = 108 days) (left) and the simulated dynamics of S when interventions were added on P, M, and S from *t* = 35 to 100 (P: − 10%, M: + 10%, S: + 10%), and on E and I from *t* = 35 to 50 (E: − 10%, I: + 10%) (right). **b** Factor “Symptom Severity and Problem Intensity”(P) of the TPQ, as empirically assessed in the real client (left) and simulated dynamics of P with the interventions as described in **a** (right). **c** Factor “Motivation to Change” (M) of the TPQ, as empirically assessed in the real client (left) and simulated dynamics of M with the interventions as described in **a** (right). **d** The dynamics of the factor “Therapeutic Alliance and Quality of the Therapeutic Relationship” of the TPQ as it was assessed in the real client (corresponding to the *b*_t_ vector) (left) and the evolution of the parameters *a, c, r, m* triggered by the dynamics of the variables and the interventions as described in **a** (right). Initial values of the variables and the parameters: E: 100; P: 79, M: 32.5, I: 50, S: 1; *a *= 0.10 (red), *c *= 0.60 (light blue), *r *= 0.35 (dark blue), *m *= 0.10 (green). Dynamic noise: 2% on E and P, 5% on M, I, S. Variables: z-transformed. Data: SIM-9, CSV-9, Patient Data: CSV-9P

Specific dynamics are shown when the *b*_*t*_-vector, which represents the empirically given dynamics of the therapeutic alliance, is introduced. Figure 9 shows the effect of interventions and of the alliance dynamics. The interventions start at *t *= 35, which realistically correspond to the treatment onset in the day treatment setting of this specific client (diagnosis: obsessive–compulsive disorder). Until that time, the client had not been involved in treatment programs because of holidays of the responsible therapist and of organizational problems at the ward. The client was disappointed, but from the moment the therapy started, she developed a good therapeutic alliance with her therapists. She was engaged in all treatments available to her, especially in a cognitive-behavioral therapy program.

## Discussion

In the described personality dynamics model of psychotherapy, a circular causality between traits and states was established. The dynamics of states—behavior, cognitions, and emotions of a client—can trigger order transitions and modify the traits. This closed circle extends the classical model of Synergetics, which focuses on the role of control parameters for the energy-driven destabilization of patterns (non-equilibrium phase transitions) onto a model of interconnected order parameters (corresponding to states) and control parameters (corresponding to traits). In psychotherapy, this circular causality conceptualizes a model of personality development and exhibits important features of psychotherapy dynamics.

### Limitations

There are some limitations in the current model and its mathematical realization. The model still contains a number of parameters shaping the various influence functions, such that they conform to a wide range of empirical knowledge about psychotherapy (see Schiepek et al. 2017). In the long run, a more minimal model should be constructed by understanding more deeply which model elements are necessary and sufficient for a particular dynamical behavior.

Another limitation concerns the question whether a model with continuous time (differential equations instead of difference equations) will also have a chaotic regime. It should be noted that the dimension of the model (D = 5) would in principle allow for chaoticity also in continuous time. For the present investigation, we decided to explore the discrete-time version of the model. Our argument here is that the dynamical variables indeed only exist at discrete time points. The process of filling out the Therapy Process Questionnaire on a daily basis goes along with a process of internal inspection, where—formally speaking—the client maps his/her complex emotional pattern to certain values of the variables. In this sense, the measurement process, induced by the TPQ, forms these variables at discrete times and the psychotherapy dynamics as a system is periodically driven by the TPQ. It is well-known that such periodic driving can trigger a complex dynamical response (Glass 2001; Hütt 2001; Hütt et al. 2002).

### Other models of psychotherapy dynamics

There are only few other attempts to mathematically model psychotherapy. Peluso et al. (2012) and Liebovitch et al. (2011) focused on the co-evolution of emotional valences expressed by a therapist and his client. The differential equations defined by the Liebovitch–Peluso–Gottman et al. group consist of segments of linear functions, each defining the gradient of emotional changes, which the client exerts on the therapist and vice versa. This leads to the prediction of stable fix-point attractors of the therapeutic relationship at the intercept of the valence functions, or to drop-outs, depending on the initial conditions in the two-dimensional phase portrait. Chaos is not possible within the scope of this model. One distinctive feature of the approach presented in this paper compared to that of the Liebovitch–Peluso–Gottman et al. group is that the current approach focuses on the psychological processes of clients in relation to their own experiences—not primarily on the client–therapist-interaction—and that we regard chaos and chaoto-chaotic phase transitions as important features of psychotherapeutic processes (Schiepek et al. 2017).

In another mathematical analysis of psychotherapeutic interventions (Haken and Tschacher 2017) the emergence of a pattern results from a competition of modes, each having a parameter value attached. The model uses a specific connectionist system (the synergetic computer), which was designed as a mathematical tool for visual pattern recognition, assuming that the scenarios of psychopathology and therapeutic interventions are analogous to that of visual pattern recognition. This approach focuses on the question under which conditions a previously established psychopathological pattern will not be restituted. One result of the simulation study is that successful corrective interventions should focus on one alternative pattern only. This alternative (healthy) pattern must be provided with higher valence (i.e., affective and motivational intensity) than the pathological pattern. The authors interpret this finding as a support of an “holistic” rather than a symptom-focused treatment approach. It is preferable to intensively support a single alternative instead of many less and only partially supported alternative patterns with less motivational intensity than the disorder. Corrective intervention must be “valent”, hence work with a focus on affective experiencing, emotion regulation, and motivation.

### Model testing

In order to test the model proposed in this paper, the time series of 941 cases (< 3% missing data in each case) are available from different psychotherapy centers, where therapy monitoring and therapy feedback by the TPQ has been implemented in routine practice for many years. A more specific empirical test on the state-trait-dynamics of the model is currently realized in the inpatient psychotherapy department of the Christian Doppler University Hospital, Salzburg, Austria. The prospective study intends to contribute to a better understanding of inter-individual variability of dynamic patterns corresponding to individual dispositions and competencies. The concrete dynamics of the variables, their initial values at the beginning of the therapeutic process, the daily input on E, I, M, P, and S as experienced by the client (interventions), and the parameter levels of *a*, *c*, *m*, and *r* (pre and post treatment) of the clients, will be assessed.

As mentioned above, the variables of the model correspond to five factors of the Therapy Process Questionnaire (Schiepek et al. 2016c), which is administered once per day in routine practice. The administration of the questionnaires is realized by an internet-based device, the Synergetic Navigation System (Schiepek et al. 2015, 2016a, c). The parameters *a*, *c*, *m*, and *r* are widely used psychological constructs, which can be assessed by known questionnaires: The parameter *a* is assessed by the “Adult Attachment Scale” (AAS, Schmidt et al. 2004) and the dynamics of the therapeutic relationship (the *b*_t_ vector of our model) by the Therapeutic Alliance Subscale of the TPQ. The parameter *c* is assessed by the “Hannover Self-Regulation Inventory” (a questionnaire on ego-functions and competencies in self–regulation; Jäger et al. 2012) and by the “Emotionale–Kompetenz–Fragebogen” (Questionnaire on Emotional Skills; Rindermann 2009). The parameter *r* is assessed by the “Essen Inventory of Resources” (Tagay et al. 2014). The parameter *m* is assessed by the “Beck Hopelessness Scale” (BHS; Beck et al. 1974; Krampen 1994) with high scores in the BHS corresponding to low levels of *m*, and by the “Questionnaire on Optimistic Expectancies on one’s Competencies” (Schwarzer 1994).

**Fig. 10:**
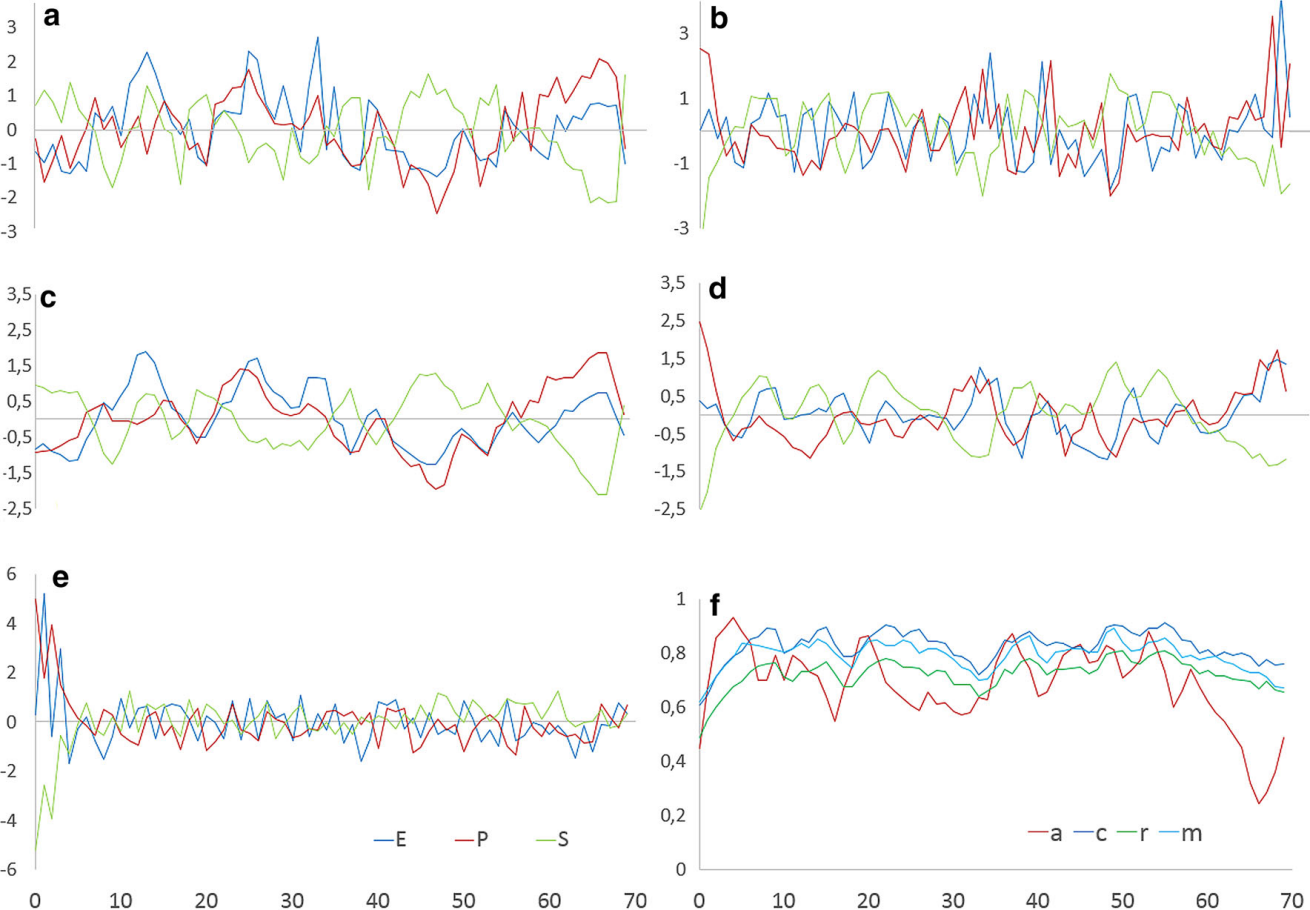
Model test by using empirical data from a real client. **a** The empirical time series of the variables E, P, and S as assessed by the TPQ. **b** Simulation of the dynamics of E, P, and S with the empirically assessed initial conditions, the *b*_*t*_ vector and the therapeutic interventions. The interventions for all variables were assessed by the client’s daily ratings of the experienced input on these variables from his environment. Below, **c**, **d** show the above time series, but smoothed by an overlapping gliding window (window width = 3, calculation of the arithmetic mean). In comparison to **b**, **e** shows the simulation run without specification of input and the *b*_t_ vector. **f** Evolution of the parameters using the *b*_t_ vector. Variables: z-transformed

Figure 10 illustrates how the model can be fitted to the specific conditions of a client, if the empirical initial conditions, the interventions as assessed by the client, and finally the quality of the relationship to her fellow clients at the ward^1^ is taken into consideration for the simulation run. The empirical data and the simulation run refer to one of our study clients, diagnosed with posttraumatic stress disorder combined with anorectic eating disorder. As can be seen, the simulation run (b) with specific information on the client taken into consideration is more similar to the empirical process (c) than the simulation run without these additional information (e): there is a slow rhythm, but no phase transition, and P and E are synchronized, whereas S is antisynchronized.

### Specific features and conclusions of our model

By summarizing the results and consequences of our mathematical model, some specific features—compared to other models (see above)—become evident:

- The option to create chaotic dynamics and chaoto-chaotic phase transitions (Kowalik et al. 1997) is an important feature of change dynamics and corresponds to empirical findings (Schiepek et al. 2016a, 2017). The model is designed in such a way that—depending on the parameters—a spectrum of dynamic patterns (e.g., chaotic patterns) occur.
- The model includes the quality of the therapeutic relationship. Findings show that the therapeutic alliance, as it is perceived by the client, correlates with and predicts the therapeutic outcome better than the alliance as perceived by the therapist or an external observer (Horvath and Symonds 1991; Orlinsky et al. 2004). The model integrates the concrete empirical dynamics of the client-therapist-relationship of a specific case and takes into consideration the evolution of the quality of cooperation as perceived by the client.
- The model does not presume the existence of alternative attractors or patterns in a potential landscape, but explains how new attractors will emerge by modulating the parameters, which are shaping the landscape. In principle, there are two complementary kinds of interventions: First, interventions can be understood as experimental inputs to explore the switching points or to identify the triggers which may switch on a different attractor within the range of unique dynamic patterns of the system. In the metaphor of potential landscapes, the ball (the realized system behavior) is driven beyond the separatrix into another valley of the landscape—if it exists. Secondly, the interventions influence the parameters via the state dynamics, and the parameters then reshape the landscape, creating new potential valleys (attractors).
- There are many ways how to create change: All variables (order parameters) of the model are open for interventions. Perhaps a converging effect of more than one component—corresponding to more than one treatment approach—is preferred. This corresponds to the well-known Dodo-Bird effect, which implies that there are no substantial differences in the effectivity of treatments (e.g., Wampold and Imel 2015).
- There might be a complementarity and synergistic effect of interventions, but without motivation to change (M) and without a positively experienced therapeutic bond, no dynamics of change will emerge. Also, our model opens the way for an evolution of M and *a* (and of other states and traits) even when a client starts from bad initial conditions.
- A long-term stabilization of treatment effects requires a change in the levels of traits (control parameters), that is, new or enhanced competencies and skills.
- There are long-term effects of psychotherapy, even after crises or rebound effects, which occur when treatments end or clients are released from inpatient or other treatment settings. Psychological long-term effects correspond to processes of neuronal reorganization which also take time and have to be stabilized even in stressful environments.
- Crises in the sense of critical instabilities are conceptualizable as important transients on the way to self-organized pattern transitions.
- The model predicts inter- and intra-individual differences in context-specific behavior depending on traits and attractors. States fluctuate depending on situational contexts (e.g., triggered by interventions) and on other states. The interconnectedness of states and traits implies that people react to situations or contexts by personal patterns of cognitions, emotions, behavior, motivation, or activation of symptoms (compare the findings of Geukes et al. 2017; Wilson et al. 2016). These patterns characterize the personality and evolve in time by self-organizing processes.

Further developments on mathematical modeling and data-related simulation of human change processes could open new ways of testing therapeutic interventions before administering them on human beings. We do not expect any options for long term predictions in chaotic systems like this, but for short term predictions and early warning signs. Conceptually, the traits of the model could be related to the ego-functions and the levels of the personality structure of clients as outlined by the Operationalized Psychodynamic Diagnostics (OPD, Doering et al. 2013). The assessment of the traits (control parameters) of the model could be cross-validated by the semi-structured interview procedures and the personality questionnaire provided by the OPD. Finally, the phenomenological (psychological) model could be more closely linked to the neural mechanisms of human change processes. Emergent psychological mechanisms could be related to more basic (meso- and micro-level) neural network dynamics (Bonzon 2017; Freeman 2000; Haken 2004; Haken and Schiepek 2010; Kozma 2016) and by this, the promising approaches of computational systems neuroscience and computational systems psychology could be integrated.

## Footnotes

1 Here, instead of the subfactor “Quality of the therapeutic relationship” of the TPQ, the subfactor “Ward atmosphere and relationship to the fellow patients” was used as vector *b*_*t*_ in Eq. 8, since the latter has been proven to be even more important than the relationship to the professionals. This is also an example of how flexible the model is in terms of testing alternative hypotheses.
